# Acidified bile acids enhance tumor progression and telomerase activity of gastric cancer in mice dependent on c‐Myc expression

**DOI:** 10.1002/cam4.999

**Published:** 2017-03-01

**Authors:** Xiaolong Wang, Lei Sun, Xijing Wang, Huafeng Kang, Xiaobin Ma, Meng Wang, Shuai Lin, Meng Liu, Cong Dai, Zhijun Dai

**Affiliations:** ^1^ Department of OncologyThe Second Affiliated Hospital of Xi'an Jiaotong UniversityXi'anShaanxi710004China; ^2^ Department of General SurgeryThe Second Affiliated Hospital of Xi'an Jiaotong UniversityXi'anShaanxi710004China

**Keywords:** Acidified bile acids, c‐Myc activation, duodenal reflux, gastric cancer, telomerase activity

## Abstract

c‐Myc overexpression has been implicated in several malignancies including gastric cancer. Here, we report that acidified bile acids enhance tumor progression and telomerase activity in gastric cancer via c‐Myc activation both in vivo and in vitro. c‐Myc mRNA and protein levels were assessed in ten primary and five local recurrent gastric cancer samples by quantitative real‐time polymerase chain reaction and western blotting analysis. The gastric cancer cell line MGC803 was exposed to bile salts (100 *μ*mol/L glycochenodeoxycholic acid and deoxycholic acid) in an acid medium (pH 5.5) for 10 min daily for 60 weeks to develop an MGC803‐resistant cell line. Control MGC803 cells were grown without acids or bile salts for 60 weeks as a control. Cell morphology, proliferation, colony formation and apoptosis of MGC803‐resistant cells were analyzed after 60 weeks. To determine the involvement of c‐Myc in tumor progression and telomere aging in MGC803‐resistant cells, we generated xenografts in nude mice and measured xenograft volume and in vivo telomerase activity. The c‐Myc and hTERT protein and mRNA levels were significantly higher in local recurrent gastric cancer samples than in primary gastric cancer samples. MGC803‐resistant cells showed a marked phenotypic change under normal growth conditions with more clusters and acini, and exhibited increased cell viability and colony formation and decreased apoptosis in vitro. These phenotypic changes were found to be dependent on c‐Myc activation using the c‐Myc inhibitor 10058‐F4. MGC803‐resistant cells also showed a c‐Myc‐dependent increase in xenograft growth and telomerase activity in vivo. In conclusion, these observations support the hypothesis that acidified bile acids enhance tumor progression and telomerase activity in gastric cancer and that these effects are dependent on c‐Myc activity. These findings suggest that acidified bile acids play an important role in the malignant progression of local recurrent gastric cancer.

## Introduction

Reflux of duodenal contents–acidified bile acids, have been proved to be involved in the pathogenesis of Barrett's esophagus 1, 2 and promote the occurrence of intestinal metaplasia and gastric cancer 3. In addition, patients with subtotal gastrectomy showed an increased risk of gastric cancer, recent studies have confirmed, reflux through the pyloric stomach, even if no previous gastric surgery can lead to gastric cancer 4. Although many studies have examined the relationship between gastric cancer incidence and duodenal reflux, few studies have evaluated the relationship between duodenal reflux and gastric cancer progression.

Bile acid deoxycholic acid (DCA) and chenodeoxycholic acid (CDCA), as the common ingredients of duodenal reflux, act synergistically in many physiological and pathological processes. For example, bile acids increase cell survival and proliferation and decrease apoptosis in vitro by activating mitogen‐activated protein kinase signaling and downregulating the caspase cascade 5 and extracellular signal‐related kinase pathway 6. Similarly, DCA and CDCA have been proved to induce the expression of transcription factors nuclear factor‐κB and caudal type homeobox‐2 for keratinocyte differentiation in esophageal keratinocytes 7, 8. Bile acids under acidified media (known as acidified bile acids) were reported to upregulate the mRNA and protein expression of the proto‐oncogene c‐Myc in Barrett's metaplasia and esophageal adenocarcinoma 9. c‐Myc gene, as a transcription factor of hTERT, was over expressed in a variety of tumors 10. It was also demonstrated that the expression of c‐Myc could induce endogenous hTERT transcription and thereby activate telomerase activity in epithelial cells and fibroblasts 1. We previously confirmed that acidified bile acids can upregulate hTERT transcription by activating c‐Myc in gastric cancer cells 11. To further confirm the in vitro effects of acidified bile acids, we used an animal model to systemically study the role of bile acids in gastric cancer. The purpose of this study was to evaluate whether acidified bile acids contribute to gastric cancer progression via c‐Myc activation.

## Materials and Methods

### Patient tissues

Real‐time PCR and Western blot analysis were performed in 10 cases of primary gastric cancer and five cases of local recurrent gastric cancer. Local recurrent gastric cancer refers to remnant gastric cancer more than 5 years after subtotal gastrectomy of gastric cancer. For the real‐time PCR, an immediate biopsy samples of RNA was extracted for processing. For Western blotting, biopsy specimens were immediately processed and stored at −20°C. Approval from the local ethics committee was granted for the study.

### Reagents

DCA, CDCA, and the c‐Myc inhibitor (10058‐F4; [Z,E]‐5‐[4‐ethylbenzylidine]‐ 2‐thioxothiazolidin‐4‐one) were purchased from Sigma‐Aldrich (St. Louis, MO) and dissolved in dimethyl sulfoxide (DMSO). Two rabbit monoclonal antibodies were used: hTERT antibody was obtained from Proteintech (Chicago, IL) and c‐Myc antibody from Santa Cruz Biotechnology (Santa Cruz, CA), respectively. Total RNA was purified using the RNeasy total RNA extraction kit (QIAGEN, Hilden, Germany) and real‐time PCR amplifications were performed with SYBR^®^ Premix Ex Taq^TM^ II (Takara, Kusatsu, Japan).

### Cell lines and cell culture conditions

MGC803 cells were purchased from the Academy of Life Science, Tongji University (Shanghai, China) and were cultured in Roswell Park Memorial Institute media supplemented with 10% fetal calf serum and 100 U/mL penicillin and 100 mg/mL streptomycin. To generate MGC803‐resistant cells, we adjusted the pH value of the MGC803 culture medium to the experimental conditions using the hydrochloric acid (A). The bile acids GCDA and DCA were diluted to optimal working concentrations of 100 *μ*mol/L (B) with culture medium, and the overall pH (A + B) was adjusted to pH 5.5, simulating the gastric environment. Initially, MGC803 cells were chronically exposed to acidified medium with bile acids (A + B) for 10 min every 24 h. The experimental time and conditions were optimized in our preliminary experiments, which showed that 10 min was enough and did not result in cell damage. This procedure was repeated and it took 60 weeks for the MGC803 cells to survive and proliferate under the exposure of A + B for 120 min 12, 13. Control untreated cells were cultured in neutral RPMI medium at pH 7.4 in parallel to the resistant cells for 60 weeks. The morphological changes in MGC803 cells exposed to acidified bile acids (A+B) were documented at 30 and 60 weeks.

### Cell proliferation assay

The growth rates of MGC803 and MGC803‐resistant cells treated with DMSO and 10058‐F4, respectively (four groups: MGC803/DMSO, MGC803/10058‐F4, MGC803‐resistant/DMSO, and MGC803‐resistant/10058‐F4), were detected by the CCK‐8 assay according to the manufacturer's protocol (CCK‐8; Dojindo Molecular Technologies Inc., Kumamoto, Japan). Briefly, 2000 cells per well were seeded into 96‐well tissue culture plates in a final 200 *μ*L volume of growth medium. After 96 h incubation, absorbance at 450 nm was measured with an Enspire Multi‐label Reader 2300 (Perkin Elmer, Waltham, MA). Each experiment was performed in triplicate.

### Colony formation assay

MGC803 and MGC803‐resistant cells were trypsinized and resuspended in growth medium, and then seeded into four‐six‐well plates (200 cells in each well). The cells were then treated with DMSO and 10058‐F4, respectively, (four groups: MGC803/DMSO, MGC803/10058‐F4, MGC803‐resistant/DMSO, and MGC803‐resistant/10058‐F4). Next, when the cultured cells grew and formed the visible cell colonies, the cells were fixed with 4% paraformaldehyde and stained with 0.1% crystal violet. The number of colonies was counted under a DM‐RBE microscope (Leica, Heidelberg, Germany). Each experiment was performed in triplicate.

### Apoptosis assay

MGC803 and MGC803‐resistant cells were seeded into six‐well plates and treated with DMSO and 10058‐F4, respectively (four groups: MGC803/DMSO, MGC803/10058‐F4, MGC803‐resistant/DMSO, and MGC803‐resistant/10058‐F4). Apoptotic analysis was using the FITC Annexin V Apoptosis Detection Kit I (BD Biosciences, Franklin Lakes, NJ) according to the manufacturer's instructions. Cells were examined by flow cytometry and the data analyses were performed using the FlowJo 7.6 software (TreeStar Inc).

### Real‐time PCR analysis

Total RNA was extracted using the RNeasy Mini Kit (Qiagen, Valencia, CA), followed by reverse transcription into cDNA with PrimeScript^™^ RT Master Mix (Takara, Kusatsu, Japan) according to the manufacturer's instructions. Then the resulting cDNA was subjected to qRT‐PCR analysis using SYBR^®^ Premix Ex Taq^™^ II (Takara, Kusatsu, Japan). Negative control, including all reagents except cDNA, was included in all runs. The primers used for amplification were showed in Table 1.

**Table 1:** Primer sequences

Gene	Sequence	PCR product
c‐Myc	5′‐ TCAAGAGGTGCCACGTCTCC ‐3′	81 bp
	5′‐ TCTTGGCAGCAGGATAGTCCTT ‐3′	
hTERT	5′‐ ATCAGACAGCACTTGAAGAG‐3′	150 bp
	5′‐ GTAGTCCATGTTCACAATCG ‐3′	
*β*‐actin	5′‐ CTTAGCACCCCTGGCCAAG ‐3′	151 bp
	5′‐ GATGTTCTGGAGAGCCCCG ‐3′	

Each reaction was performed in a final volume of 25 *μ*L containing 2.0 *μ*L of appropriately diluted cDNA, 0.5 *μ*L (10 *μ*mol/L) of forward and reverse primers specific for human c‐Myc, hTERT or *β*‐actin, 12.5 *μ*L of SYBR Premix Ex Taq, and 9.5 *μ*L of water. The qPCR reaction mixtures were denatured at 94°C for 3 min followed by 40 cycles at 94°C for 30 sec, 51°C for 30 sec (c‐Myc), or 61°C for 30 sec (hTERT), and 72°C for 60 sec. Each reaction was performed in triplicate and the mean mRNA level in each cell line was calculated by the 2^−ΔΔCt^ method.

### Western blot analysis

All gastric cancer biopsies were taken from a −80°C tissue bank and animal tumor tissues were from subcutaneous transplanted xenografts in nude mice. All protein samples were homogenized on ice in lysis buffer containing 40 mmol/L Tris‐HCl (pH 6.9), 2 mmol/L ethylenediaminetetraacetic acid (EDTA, pH 8.0), 100 mmol/L sodium fluoride, 150 mmol/L NaCl, 10 mmol/L sodium pyrophosphate, 1% Tergitol type NP‐40, 2 mmol/L sodium orthovanadate, 1% Triton X‐100, 1.0 mmol/L phenylmethanesulfonyl fluoride (PMSF), and 1 × protein inhibitor mini‐tablet (Roche, Basel, Switzerland). Lysates were then clarified by centrifugation at 12000 *g* for 10 min at 4°C. Protein concentration was estimated using the Pierce Protein Estimation System (Thermo Fisher Scientific, Waltham, MA) according to the manufacturer's protocol. Next, equal amounts (30 *μ*g) of protein were heated at 95°C for 5 min in 5 × Laemmli sample buffer and then separated on a 10% SDS‐PAGE gel and transferred onto polyvinylidene difluoride (PVDF) membranes by wet transfer method. Nonspecific binding was blocked for 1 h at 37°C using 10% fat‐free milk in TBS containing 0.1% Tween‐20. Membranes were probed overnight at 4°C with primary antibodies (rabbit anti‐c‐Myc, 1:1000, Santa Cruz Biotechnology; rabbit anti‐ *β* ‐actin, 1:1000 dilution, Sigma‐Aldrich; and rabbit anti‐hTERT, 1:1000 dilution; Proteintech). After washing three times with TBST, horseradish peroxidase–conjugated secondary antibodies (1:5000 dilution; Proteintech Group) were then added for incubations at 27°C for 1 h. Immunoreactive bands were visualized using SuperSignal West Femto Maximum Sensitivity Substrate (Thermo Fisher Scientific Inc., Waltham, MA) and exposed using the ChemiDoc XRS+ (Bio‐Rad, Hercules, CA).

### Tumorigenicity assay

Four‐week‐old BALB/c nude mice were purchased from the Center of Laboratory Animals, Peking University Health Science Center (Beijing, China), and the experiment was performed following the Guide to Management and Use of Experimental Animals, National Science Council, China. Tumors were established by subcutaneous inoculation with 5 × 10^6^ cells (four groups: MGC803/DMSO, MGC803/10058‐F4, MGC803‐resistant/DMSO, and MGC803‐resistant/10058‐F4) into one side of the axilla of six mice per group. Tumor volume was calculated using the empirical formula *V* = 0.52 × [(shortest diameter)^2^ × (longest diameter)]. When tumor volumes reached 100 mm^3^, two groups of mice with MGC803‐resistant xenografts were intravenously given 10058‐F4 (30 mg/kg/dose) daily for 8 days (from the third to tenth day after tumor transplantation) 14, 15. After 11 days, all mice were killed by cervical dislocation, followed by the tumor tissues dissecting and weighing. Tumor specimens were subjected to histopathological analysis and telomerase activity assays. The animal procedures were approved by the Experimental Animal Center of Xi'an Jiaotong University Health Science Center.

### Telomerase activity assay

Telomerase activity assay was determined by the stretch PCR method using a telomerase activity detection kit (TeloChaser; Toyobo Co. Ltd., Osaka, Japan) according to the manufacturer's instructions. Briefly, xenograft tissues from nude mice were pelleted, washed in ice‐cold PBS, homogenized in 200 *μ*L ice‐cold lysis buffer, and incubated on ice for 30 min. The cellular lysate was centrifuged at 12,000*g* for 20 min at 4°C to remove the insoluble fraction and the supernatant was collected and stored at −80°C. Nine micrograms of protein was used for PCR detection. After incubating at 37°C to produce telomeric repeats for 60 min, the DNA products were then isolated, heated at 90°C for 3 min and subjected to 30 cycles of PCR including 94°C for 30 sec, 50°C for 30 sec, and 72°C for 60 sec. The PCR products were electrophoresed on a 10% polyacrylamide gel and stained with ethidium bromide. Images were captured using the FLA___3000G image analyzer (Fuji Film Corp., Tokyo, Japan).

### Statistical analyses

The results are shown as the means ± standard error. Differences were analyzed using unpaired two‐tailed Student's *t*‐tests with unequal variance for multiple comparisons by SPSS software 19.0 (SPSS, Inc., Chicago, IL). Two‐sided *P *< 0.05 were reported as significant. All experiments were repeated independently at least three times.

## Results

### c‐Myc and hTERT expression in primary and local recurrent gastric cancer

**Figure 1:**
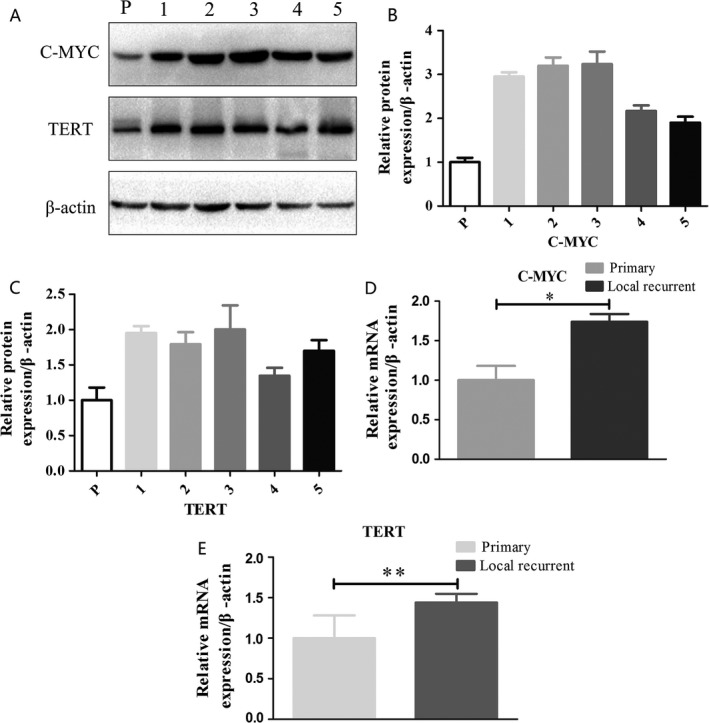
c‐Myc and hTERT expression in primary gastric cancer and local recurrent gastric cancer samples. (A) Western blot analysis of c‐Myc and hTERT protein expression in primary gastric cancer (P: average protein expression in primary gastric cancer samples) and local recurrent gastric cancer samples (1, 2, 3, 4, and 5). (B) Integrated optical density was measured to evaluate c‐Myc protein expression relative to *β*‐actin. (C) Integrated optical density was measured to evaluate hTERT protein expression relative to *β*‐actin. (D) Real‐time PCR of c‐Myc mRNA expression in primary gastric cancer (Primary: average mRNA expression in primary gastric cancer samples) and local recurrent gastric cancer samples (Local recurrent) (*P < 0.05). (E) Real‐time PCR of hTERT mRNA expression in primary gastric cancer (Primary: average mRNA expression in primary gastric cancer samples) and local recurrent gastric cancer samples (Local recurrent) (**P < 0.05).

To compare c‐Myc and hTERT protein and mRNA expression, we selected 15 samples; 10 from patients with primary gastric cancer and five from patients with local recurrent gastric cancer, which refers to remnant cancer after subtotal gastrectomy of gastric cancer. c‐Myc expression was detected in all gastric cancer samples (Fig. 1A), demonstrating higher c‐Myc protein levels in the five local recurrent gastric cancer samples compared to in the primary gastric cancer samples. All c‐Myc immunoreactive bands were measured by densitometry, and the average c‐Myc protein levels in primary gastric cancer and local recurrent gastric cancer are shown in Figure 1B (all *P *< 0.05). In real‐time PCR, we observed upregulation of c‐Myc mRNA in local recurrent gastric cancer samples compared to in primary gastric cancer samples (Fig. 1D, *P *< 0.05). In the samples showing upregulation of c‐Myc, the median increase was 1.74‐fold between the local recurrent gastric cancer group and primary gastric cancer group.

The expression of hTERT in the two groups was the same as that of c‐Myc; hTERT expression was observed in all samples. Western blotting and real‐time PCR analysis of hTERT showed lower expression in the primary gastric cancer group compared to in the local recurrent gastric cancer group (Fig. 1C and E). The median increase in hTERT mRNA expression was 1.44‐fold between the local recurrent gastric cancer group and primary gastric cancer group (*P *< 0.05). These results indicate that the bile acid reflux environment after gastrectomy causes increased expression of c‐Myc and hTERT.

### Generation of MGC803 cells resistant to acidified bile acids

To simulate chronic local recurrent disease in vitro, the gastric cancer cell line MGC803 was exposed to acidified medium (pH 5.5) containing 100 *μ*mol/L DCA and CDCA. An untreated log‐growth MGC803 cell line was generated to be used as a control in normal pH media. After daily 10‐min exposure to the acidified bile acids for 60 weeks, MGC803‐resistant cells were able to survive and proliferate after 120‐min exposure.

### Effects of acidified bile acids on MGC803 cell morphology, viability, colony formation, and apoptosis

**Figure 2:**
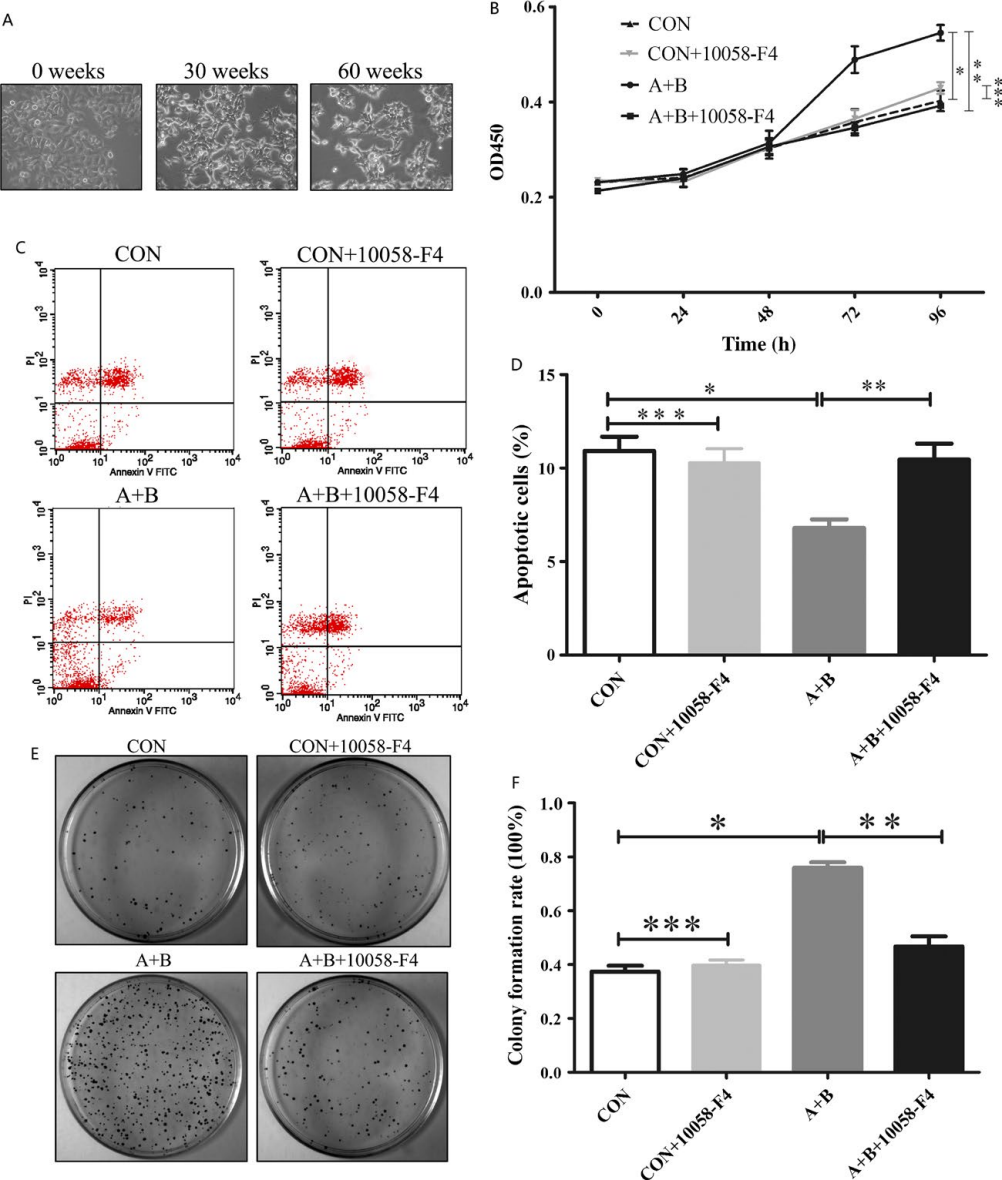
Acidified bile acids promote a malignant phenotype in MGC803 cells dependent on c‐Myc activity in vitro. (A) Morphological changes in MGC803 cells following treatment with acidified bile acids (A+B) for different durations. (B) Cell viability of MGC803 cells (CON), MGC803 cells/10058‐F4 (c‐Myc inhibitor; CON+10058‐F4), MGC803‐resistant cells (A+B), and MGC803‐resistant cells/10058‐F4 (A+B+10058‐F4) by the CCK‐8 assay. (C) Flow cytometry apoptosis assay. (D) Apoptosis rates of MGC803 cells, MGC803 cells/10058‐F4, MGC803‐resistant cells, and MGC803‐resistant cells/10058‐F4). (E) Colony formation assay. (F) Colony formation rates of MGC803 cells, MGC803 cells/10058‐F4, MGC803‐resistant cells, and MGC803‐resistant cells/10058‐F4). (*P < 0.05: MGC803 cells vs. MGC803‐resistant cells; **P < 0.05: MGC803‐resistant cells/DMSO vs. MGC803‐resistant cells/10058‐F4; ***P > 0.05: MGC803 cells versus MGC803 cells/10058‐F4).

To determine the effect of acidified bile acids on gastric cancer cells in vitro, we examined the changes in morphology, cell viability, colony formation, and apoptosis in the four cell groups (MGC803/DMSO, MGC803/10058‐F4, MGC803‐resistant/DMSO, and MGC803‐resistant/10058‐F4). Morphological changes were evident between untreated MGC803 cells and MGC803‐resistant cells from 30 weeks onward. However, changes in the different phenotypes were observed at 60 weeks (Fig. 2A). MGC803‐resistant cells, like an oval cells, showed alveolar formation, and MGC803 cells kept fusiform, uniformly dispersed on the dish. The viabilities of the four groups of cells were assessed using the CCK‐8 assay. MGC803‐resistant cells showed better cell viability compared to MGC803 cells (*P < 0.05, Fig. 2B). In addition, the c‐Myc inhibitor 10058‐74 decreased the viability of MGC803‐resistant cells, but not the MGC803 cells (**P < 0.05, ***P > 0.05, Fig. 2B). Moreover, apoptosis assays showed that the rate of apoptosis was decreased in MGC803‐resistant cells compared to in MGC803 cells (*P < 0.05, Fig. 2C and D). In contrast, the c‐Myc inhibitor 10058‐74 decreased the MGC803‐resistant cell apoptosis rate, but not the MGC803 cells (**P < 0.05, ***P > 0.05, Fig. 2C and D). Colony formation assays also showed a significant induction in the numbers of colonies formed by MGC803‐resistant cells (*P < 0.05, Fig. 2E and F). The c‐Myc inhibitor 10058‐74 decreased the induction colony formation in MGC803‐resistant cells, but not the MGC803 cells (**P < 0.05, ***P > 0.05, Fig. 2E and F). These results indicate that acidified bile acids induce morphological changes in gastric cancer cells and increase their viability and colony formation ability and decrease apoptosis rates through a c‐Myc‐dependent mechanism in vitro.

### Comparing xenograft formation and growth between MGC803 cells and MGC803‐resistant cells in nude mice

**Figure 3:**
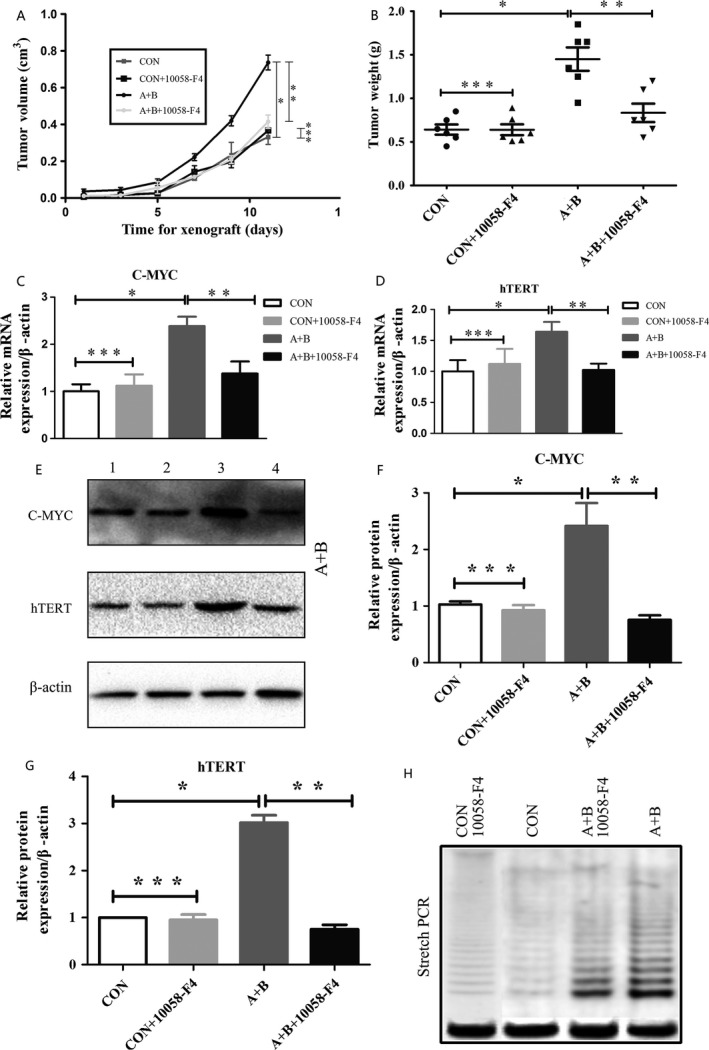
Acidified bile acids promote MGC803 xenograft formation and telomerase activity dependent on c‐Myc activity in vivo. (A) Growth curves of MGC803 (CON), MGC803+10058‐F4 (CON+10058‐F4), MGC803‐resistant (A+B), and MGC803‐resistant/10058‐F4 (A+B+10058‐F4) xenografts. (B) Tumor weight of MGC803 (CON), MGC803+10058‐F4 (CON+10058‐F4), MGC803‐resistant (A+B), and MGC803‐resistant/10058‐F4 (A+B+10058‐F4) xenografts. (C) Real‐time PCR of c‐Myc mRNA expression in MGC803 (CON), MGC803+10058‐F4 (CON+10058‐F4), MGC803‐resistant (A+B), and MGC803‐resistant/10058‐F4 (A+B+10058‐F4) xenografts. (D) Real‐time PCR of hTERT mRNA expression in MGC803 (CON), MGC803+10058‐F4 (CON+10058‐F4), MGC803‐resistant (A+B), and MGC803‐resistant/10058‐F4 (A+B+10058‐F4) xenografts. (E) Western blot analysis of c‐Myc and hTERT protein expression in MGC803 (CON), MGC803+10058‐F4 (CON+10058‐F4), MGC803‐resistant (A+B), and MGC803‐resistant/10058‐F4 (A+B+10058‐F4) xenografts. (F) Integrated optical density was measured to evaluate c‐Myc protein expression relative to *β*‐actin. (G) Integrated optical density was measured to evaluate hTERT protein expression relative to *β*‐actin. (H) Telomerase activity in MGC803 (CON), MGC803+10058‐F4 (CON+10058‐F4), MGC803‐resistant (A+B), and MGC803‐resistant/10058‐F4 (A+B+10058‐F4) xenografts by stretch PCR. (*P < 0.05: MGC803 cells vs. MGC803‐resistant cells; **P < 0.05: MGC803‐resistant cells/DMSO versus MGC803‐resistant cells/10058‐F4; ***P > 0.05: MGC803 cells versus MGC803 cells/10058‐F4). DMSO, dimethyl sulfoxide.

To investigate the effects of acidified bile acids on the formation of gastric cancer xenografts in vivo, MGC803 cells and MGC803‐resistant cells were injected into the axilla of nude mice. We found that MGC803‐resistant cells exhibited faster growth in BALB/c nude mice compared to control MGC803 cells (*P < 0.05, Fig. 3A and B). Moreover, our results showed that the growth of MGC803‐resistant xenografts was significantly inhibited compared to control MGC803 xenografts after injecting 10058‐F4 (**P < 0.05, ***P > 0.05, Fig. 3A and B).

To investigate the molecular events associated with the effects of acidified bile acids on gastric cancer progression, we assessed whether acidified bile acids affected c‐Myc and hTERT expression in the MGC803 xenografts. c‐Myc and hTERT protein expression was detected in all four groups (MGC803 xenografts/DMSO, MGC803 xenografts/10058‐F4, MGC803‐resistant xenografts/DMSO, and MGC803‐resistant xenografts/10058‐F4) at the mRNA and protein levels. Although c‐Myc and hTERT showed higher protein expression in MGC803‐resistant xenografts than in MGC803 xenografts, their expression was significantly downregulated under 10058‐F4 treatment (Fig. 3E–G). The changes in mRNA expression mirrored the protein expression changes (Fig. 3C and D). These results indicate that acidified bile acids increase gastric cancer cell xenograft formation and growth and induced c‐Myc‐dependent hTERT expression in vivo.

### Induction of telomerase activity by acidified bile acids via c‐Myc activation

To explore the effects of acidified bile acids on the aging of gastric cancer cell xenografts, we assessed telomerase activity in MGC803 and MGC803‐resistant xenografts. Telomerase activity was measured by stretch PCR and expressed as a ladder of 6‐bp bands or multiples of 6‐bp intervals. Our results showed that telomerase activity in MGC803‐resistant xenografts was significantly higher than in MGC803 xenografts, and 10058‐F4 inhibited this trend (Fig. 3H). The percentage induction of telomerase was calculated using the band intensity (Table 2.), and these results suggest that acidified bile acids upregulate telomerase activity by activating c‐Myc in gastric cancer.

**Table 2:** The relative activity of telomerase in different cells

Groups	Telomere activity intensity ratio	*t* value	*P* value
CON+10058‐F4	1.101 ± 0.058	2.563^c^	0.0625
CON	1.000 ± 0.036		
A+B+10058‐F4	2.053 ± 0.054	9.682^b^	0.0006
A+B	2.534 ± 0.067	34.93^a^	0.0001

a CON versus A+B.

b A+B versus A+B+10058‐F4.

c CON versus CON+10058‐F4.

## Discussion

We hypothesized that prolonged repeated exposure of MGC803 cells to acidified bile acids would promote malignancy. Indeed, we found that MGC803‐resistant cells showed progressive morphological, molecular, and biological changes associated with a more malignant phenotype. These changes were observed as enhanced cell viability, colony formation, xenograft formation, telomerase activity, and decreased apoptosis capacity, which were dependent on c‐Myc expression. Our results show that c‐Myc and hTERT expression in local recurrent gastric cancer tissues was much higher than in primary gastric cancer tissues at the protein and mRNA levels. Furthermore, we showed that acidified bile acids induced hTERT overexpression in human gastric cancer cells through c‐Myc activation, suggesting that acidified bile acids promote tumor progression and telomerase activity via c‐Myc activation.

Reflux of duodenal contents was found to be closely associated with the development and proliferation of gastric adenocarcinoma 16, 17, 18, but the mechanism remains unclear. The unconjugated bile acids DCA and CDCA, as the main component of duodenal reflux, has been extensively studied in cell culture models. Moreover, it has been confirmed that the expression of c‐myc has been induced by acidified bile acids 9. Because c‐Myc is a key factor in the development and aging of gastric cancer 19, we hypothesized that acidified bile acids influence the development and aging of gastric cancer via c‐Myc expression. In this study, MGC803‐resistant cells showed enhanced cell viability, colony formation, xenograft formation, telomerase activity, and decreased apoptosis, which were dependent on c‐Myc activity.

In local recurrent gastric cancer patients, reflux is inherently an intermittent event, which we attempted to mimic in vitro. Unlike continuous acidified bile acid exposure, chronic short exposure resulted in different outcomes to the stimulation, which may be critical in terms of biological response. In this study, we compared the gastric cancer cell line MGC803 with bile acid‐resistant MGC803 cells to determine the major changes induced by chronic short‐exposure treatment with acidified bile acids. The cells were repeatedly exposed to 100 *μ*mol/L CDCA and DCA at pH 5.5 for up to 120 min. These exposure concentration and time was the average levels of local recurrent gastric cancer patients, as these patients underwent subtotal gastrectomy, resulting in longer time and higher concentration duodenal reflux compared to normal individuals.

Occurrence of gastric cancer involves many key cellular processes, including apoptosis, proliferation, and anchorage‐dependent growth 20, 21. In general, these processes are strictly regulated by many factors, including the proto oncogene c‐Myc. c‐Myc gene is located in chromosome region 8q23‐24 and recent gastric cancer research showed that high expression of c‐Myc gene could promote cell proliferation and predict poor prognosis of patients with gastric cancer 22. The role of c‐Myc gene in gastric cancer tissues, however, is not so simple, and its precise regulation of cell proliferation, cell arrest and apoptosis depends on the types of organization and environment 23. No integrated research of c‐Myc protein expression under the treatment of acidified bile acids in gastric adenocarcinoma have been reported. In this study, we demonstrated that acidified bile acids induced tumor progression in human gastric cancer cells through c‐Myc activation. In our study, we observed that the precise mechanism of acidified bile acids‐mediated tumor progression, can be a very complex events involving a variety of signaling proteins and pathways 24. Several complex studies have shown that acid‐mediated effects on cellular proliferation involves many factors such as activation of protein kinase C and mitogen‐activated protein kinase pathways 25, whereas bile salt‐induced proliferation is mediated by epidermal growth factor receptor and farnesoid X receptor signaling 26. Further studies are required to determine whether these pathways are instrumental in regulating the acidified bile acid‐mediated proliferation observed in this study.

The telomerase subunit, which is highly expressed in normal cells and cells that continuously divide beyond replicative senescence, plays a decisive role in the telomerase activity 27. Several in vivo and in vitro studies have clearly demonstrated that hTERT is a determinant of telomerase activity 28. Because acidified bile acids have been reported to enhance c‐Myc expression 9, acidified bile acids may also enhance hTERT expression and telomerase activity, thus increasing the proliferative capacity of gastric cancer cells. We demonstrated that acidified bile acids induced hTERT overexpression and telomerase activity in human gastric cancer cells and that inhibition of c‐Myc heterodimerization by 10058‐F4 29 significantly decreased acidified bile acid‐induced hTERT expression. This suggests that acidified bile acids upregulate hTERT expression and telomerase activity through c‐Myc. Interestingly, we previously observed that acidified bile acids upregulated hTERT transcription by enhancing the binding of c‐Myc to the hTERT promoter 11. It is possible that acidified bile acids also indirectly regulate hTERT transcription through c‐Myc 30. The mechanisms through which acidified bile acids induce c‐Myc expression require further analysis.

In conclusion, we demonstrated that local recurrent gastric cancer patients showed higher c‐Myc and hTERT expression at the protein and mRNA levels. Our findings suggest that acidified bile acids induce tumor progression and telomerase activity in gastric cancer both in vivo and in vitro.

## Conflict of Interest

None declared.
